# Genomic and phenotypic profiling of *Staphylococcus aureus* isolates from bovine mastitis for antibiotic resistance and intestinal infectivity

**DOI:** 10.1186/s12866-023-02785-1

**Published:** 2023-02-20

**Authors:** Satwik Majumder, Trisha Sackey, Charles Viau, Soyoun Park, Jianguo Xia, Jennifer Ronholm, Saji George

**Affiliations:** 1 Department of Food Science and Agricultural Chemistry, Macdonald Campus, McGill University, Macdonald-Stewart Building, Room-1039, 21, 111 Lakeshore Ste Anne de Bellevue, Quebec, H9X 3V9 Canada; 2 Institute of Parasitology, Macdonald Campus, McGill University, 21, 111 Lakeshore Ste Anne de Bellevue, Quebec, H9X 3V9 Canada; 3 Department of Animal Science, Macdonald Campus, McGill University, 21, 111 Lakeshore Ste Anne de Bellevue, Quebec, H9X 3V9 Canada

**Keywords:** *Staphylococcus aureus*, Bovine mastitis, Antibiotics, Antibiotic resistance (ABR), Virulence characteristics, Intracellular pathogens, Zoonotic spillover, Intestinal infection, Caco-2 cells, *Caenorhabditis elegans*

## Abstract

**Background:**

*Staphylococcus aureus* is one of the prevalent etiological agents of contagious bovine mastitis, causing a significant economic burden on the global dairy industry. Given the emergence of antibiotic resistance (ABR) and possible zoonotic spillovers, *S aureus* from mastitic cattle pose threat to both veterinary and public health. Therefore, assessment of their ABR status and pathogenic translation in human infection models is crucial.

**Results:**

In this study, 43 *S. aureus* isolates associated with bovine mastitis obtained from four different Canadian provinces (Alberta, Ontario, Quebec, and Atlantic provinces) were tested for ABR and virulence through phenotypic and genotypic profiling. All 43 isolates exhibited crucial virulence characteristics such as hemolysis, and biofilm formation, and six isolates from ST151, ST352, and ST8 categories showed ABR. Genes associated with ABR (*tetK, tetM, aac6’, norA, norB, lmrS*, *blaR, blaZ*, etc.), toxin production (*hla, hlab*, *lukD*, etc.), adherence (*fmbA, fnbB, clfA, clfB*, *icaABCD*, etc.), and host immune invasion (*spa, sbi, cap, adsA*, etc.) were identified by analyzing whole-genome sequences. Although none of the isolates possessed human adaptation genes, both groups of ABR and antibiotic-susceptible isolates demonstrated intracellular invasion, colonization, infection, and death of human intestinal epithelial cells (Caco-2), and *Caenorhabditis elegans*. Notably, the susceptibilities of *S. aureus* towards antibiotics such as streptomycin, kanamycin, and ampicillin were altered when the bacteria were internalized in Caco-2 cells and *C. elegans*. Meanwhile, tetracycline, chloramphenicol, and ceftiofur were comparatively more effective with ≤ 2.5 log_10_ reductions of intracellular *S. aureus*.

**Conclusions:**

This study demonstrated the potential of *S. aureus* isolated from mastitis cows to possess virulence characteristics enabling invasion of intestinal cells thus calling for developing therapeutics capable of targeting drug-resistant intracellular pathogens for effective disease management.

**Supplementary Information:**

The online version contains supplementary material available at 10.1186/s12866-023-02785-1.

## Background

*Staphylococcus aureus* is a versatile pathogen that is reported to cause a plethora of infections ranging from superficial skin infections to life-threatening diseases in livestock [1]. It is considered to be the most prevalent etiological agent of contagious bovine mastitis worldwide causing a significant economic burden on the dairy industry [2]. The prevalence of bacterial infections is not only a challenge for the clinical management of mastitis but could also be a public health concern as mastitis may contribute to zoonotic spillover (the transfer of bacteria or genetic determinants to humans) majorly through direct contact with the infected dairy cattle [3]. In humans, *S. aureus* has been associated with a wide range of infectious diseases such as endocarditis, hemolytic pneumonia, toxin-mediated conditions such as scalded skin syndrome, staphylococcal food poisoning or gastroenteritis, toxic shock syndromes, etc. [4].

In contrast to environmental pathogens, infections caused by contagious mastitis pathogens such as *S. aureus* are difficult to treat in agricultural settings due to an array of virulence traits including biofilm formation, toxin and enzyme production, evasion of phagocytic/non-phagocytic host cells, and immune defense mechanisms [5]. *S. aureus* can switch its phenotypes between wild types and small colony variants, and survive intracellularly contributing to persistent colonization in the intramammary environment, leading to recurrent bovine mastitis [6]. The persistence of such infection within the mammary gland demands the frequent use of antibiotics, the excessive application of which over the years has led to antibiotic resistance (ABR) and treatment failures [7]. Antibiotics including aminoglycosides, ß-lactams, cephalosporins, tetracyclines, *etc*. have been used for controlling *S. aureus* infection, however, these antibiotics have shown inconsistent efficiency [8].

Although there is plenty of information on the infectivity of *S. aureus* from cattle in mastitis infection models, there are prominent knowledge gaps on the pathogenic translation of mastitic *S. aureus* in human infection models. Human intestinal epithelial Caco-2 cells are considered to be an efficient model to investigate bacterial intracellular invasion, while *Caenorhabditis elegans* are broadly accepted as an *in vivo* model with higher throughput to assess bacterial intestinal pathogenicity and antimicrobial efficacy [9]. The acceptability of *C. elegans* is based on the key similarities with the mammalian intestine such as the presence of polarized epithelial cells with microvilli, and the first line of defense against invading pathogens [10].

In this study, we examined 43 *S. aureus* isolates collected from Canadian dairy cattle with active mastitis for ABR and virulence characteristics through genotypic and phenotypic profiling. These isolates were chosen as a subpopulation of the entire collection available at the Mastitis Pathogen Culture Collection (MPCC). We investigated the infectivity of the isolates in intestinal infection models of Caco-2 cells and *C. elegans* and assessed the efficiency of antibiotics in remediating such infections. Additionally, the whole genome analysis data available for the selected isolates were used to compare the genotype and phenotype for ABR and virulence characteristics.

## Results

### Prevalence of antibiotic resistance in *S. aureus* isolates

**Fig. 1:**
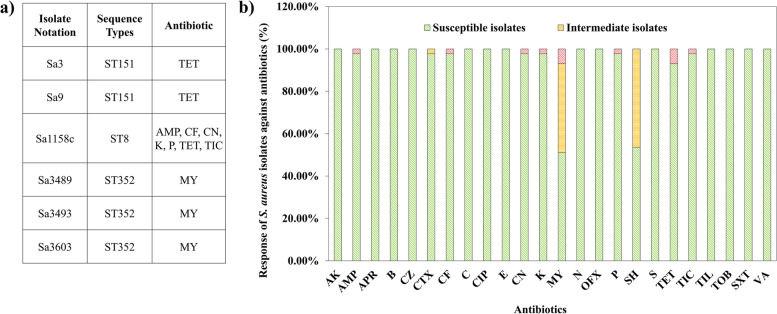
Response of 43 *S. aureus* isolates against antibiotics. **a** List of antibiotic-resistant isolates. **b** Bacterial responses toward 24 antibiotics. The *S. aureus* isolates were subjected to Kirby-Bauer disc diffusion susceptibility tests. The scores based on CLSI guidelines for susceptibility or resistance to an antibiotic were generated for each isolate. Abbreviations used—AK: Amikacin; AMP: Ampicillin; APR: Apramycin; B: Bacitracin; CZ: Cefazolin; CTX: Cefotaxime; CF: Ceftiofur; C: Chloramphenicol; CIP: Ciprofloxacin; E: Erythromycin; CN: Gentamycin; K: Kanamycin; MY: Lincomycin; N: Neomycin; OFX: Ofloxacin; P: Penicillin; SH: Spectinomycin; S: Streptomycin; TET: Tetracycline; TIC: Ticarcillin; TIL: Tilmicosin; TOB: Tobramycin; SXT: Trimethoprim/Sulfamethoxazole; VA: Vancomycin. The experiment was performed in triplicates and repeated thrice to ensure reproducibility

Out of the 43 isolates, 6 isolates showed either single antibiotic resistance (5/6) or multi-antibiotic resistance (1/6) (Fig. 1a and Table S3a). The isolates Sa3 and Sa9 showed resistance to tetracycline, whereas isolates Sa3489, Sa3493, and Sa3603 were resistant to lincomycin. Isolate Sa1158c showed resistance to multiple classes of antibiotics including ampicillin, gentamycin, kanamycin, penicillin, tetracycline, ticarcillin, and ceftiofur. Intermediate responses to lincomycin and spectinomycin were observed from 39.5% and 46.5% of the isolates, respectively (Fig. 1b). Eight different sequence types (ST) covering the 43 isolates were identified where 34 of the isolates either belonged to ST151 or ST352 (Table S1). The two tetracycline-resistant and three lincomycin-resistant isolates were from ST151 and ST352 respectively, whereas the multi-drug-resistant isolate belonged to ST8. Clinically important ABR genes were identified from the whole genome data. For instance, tetracycline-resistant genes (*tet(K)*; 1/43, *tet(M)*; 3/43 and *tet(38)*; 43/43), lincomycin-resistant genes (*lnu(A)*; 3/43), aminoglycoside-resistant genes (*aac(6')*; 1/43, *aph(3')*; 43/43, *aac3*; 43/43), ß-lactam and cephalosporin-resistant genes (*blaI, blaR, blaZ*; 1/43, *mecA*; 1/43), and multi-drug resistant regulators (*arlR, arlS, mgrA*; 43/43) were evident.

**Fig. 2:**
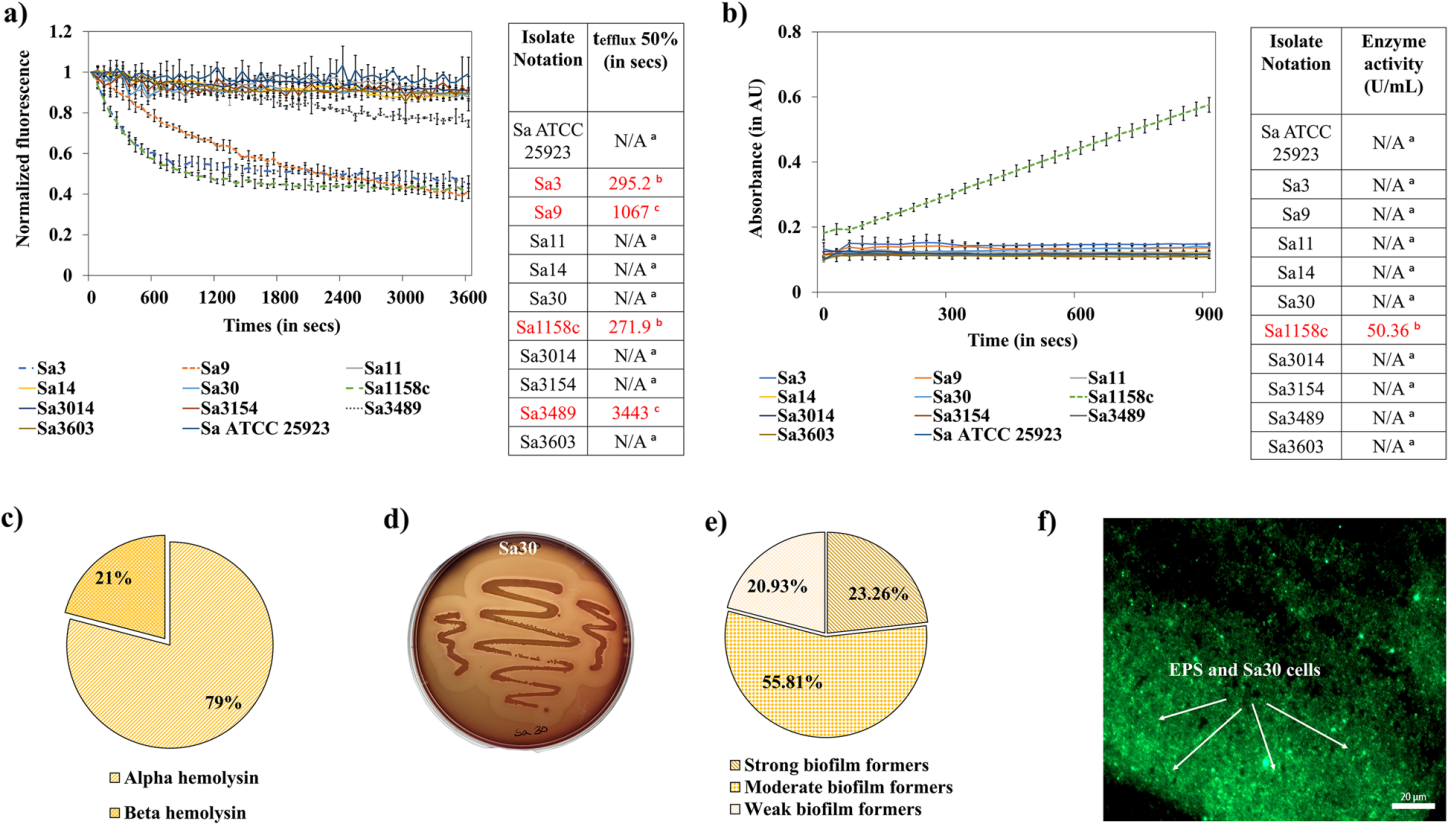
Antibiotic-resistance mechanisms and virulence factors in *S. aureus* isolates. **a** Assessment of efflux pump activity in five antibiotic-resistant and five susceptible isolates. EtBr efflux assay was performed using 3 µg/mL of EtBr and 30 µg/mL of CPZ. The fluorescent intensity (530 nm/590 nm) was monitored for 60 min after reenergizing the bacterial cells to trigger EtBr efflux with glucose (0.4% v/v). **b** Assessment of ß-lactamase enzyme activity in the isolates. The isolates were subjected to a Nitrocefin assay where the absorbance of the cell-free extract mixed with Nitrocefin was detected at 490 nm for 15 min. **c** Distribution of hemolysin manifestation by the 43 isolates. **d** Alpha hemolysin manifestation by Sa30. Each isolate was cultured in TSA plates with 5% sheep blood for 24 h. The hemolysis was detected visually by the translucency around the bacterial colony. **e** Distribution of biofilm-forming ability by the 43 isolates. The biofilm formation was assessed using a crystal violet assay. All isolates were classified into weak, moderate, and strong biofilm-formers based on their biofilm-forming ability. **f** Fluorescence microscopic image of extracellular polymeric substances (EPS) and Sa30 cells. A green (505 nm) filter was used to acquire the GFP-labeled Sa30 biofilms using a high-content screening microscope, Cell Discoverer 7. Alphabets (in tabular data) indicate a significant difference (*p* < 0.05). ‘N/A’ stands for not applicable. More data on the resistance mechanisms and virulence factors are provided in Tables S3 and S5. The experiment was performed in quadruplicates and repeated thrice to ensure reproducibility

EtBr efflux assay and Nitrocefin assay were performed to assess efflux pump activity and ß-lactamase enzyme activity, respectively in all the isolates (Table S3b). The tetracycline-resistant Sa3 and Sa9, lincomycin-resistant Sa3489, and the multi-drug-resistant Sa1158c showed active efflux pump activity. For instance, isolates Sa3, Sa9, Sa3489, and Sa1158c extruded 50% of the EtBr in 295.2 sec, 1067 sec, 3443 sec, and 271.9 sec, respectively (Fig. 2a, see the tabular data). The ß-lactamase enzyme activity was observed only in the multi-drug resistant isolate (50.36 U/mL) (Fig. 2b, see the tabular data). Genome analysis indicated the presence of genes associated with MFS efflux pumps (*norA*; 43/43, *norB*; 43/43, *lmrS*; 43/43, *tet(38)*; 43/43, *tet(K); 1/43, tet(M)*; 3/43), MATE efflux pumps (*mepR*, *mepA*, *mepB*; 43/43), and ß-lactamase enzyme activity (*blaI, blaR, blaZ*; 1/43). The list of genes associated with antibiotic resistance is provided in Tables 1 and S4.

**Table 1:** List of the genes associated with antibiotic resistance and adherence in the six antibiotic-resistant isolates (Sa3, Sa9, Sa1158c, Sa3489, Sa3493, and Sa3603), and five antibiotic-susceptible isolates (Sa11, Sa14, Sa30, Sa3014, and Sa3154)

Isolate ID	Isolate notation	Sequence Types	Antibiotic-resistant genes	Virulence genes			
				**Clumping factor**	**Fibronectin binding proteins**	**Intercellular adhesin**	**Staphylococcal protein A**
30704176	Sa3	ST151	aac3, aph(3'), arlR, arlS, dhaP, lmrS, mepA, mepB, mepR, mgrA, norA, norB, rlmH, tet38, tetK	clfB	xx	icaA, icaB, icaC, icaD, icaR	xx
41704653	Sa9	ST151	aac3, aph(3'), arlR, arlS, dhaP, lmrS, mepA, mepB, mepR, mgrA, norA, norB, rlmH, tet38	clfA, clfB	xx	icaA, icaB, icaC, icaD, icaR	xx
32200324	Sa11	ST352	aac3, aph(3'), arlR, arlS, dhaP, lmrS, mepA, mepB, mepR, mgrA, norA, norB, rlmH, tet38	clfA, clfB	fnbA, fnbB	icaA, icaB, icaC, icaD, icaR	spa
22200587	Sa14	ST3028	aac3, aph(3'), arlR, arlS, dhaP, lmrS, mepA, mepB, mepR, mgrA, norA, norB, rlmH, tet38	clfA	fnbA, fnbB	icaA, icaB, icaC, icaD, icaR	spa
21000024	Sa30	ST352	aac3, aph(3'), arlR, arlS, dhaP, lmrS, mepA, mepB, mepR, mgrA, norA, norB, rlmH, tet38	clfA, clfB	fnbA, fnbB	icaA, icaB, icaC, icaD, icaR	spa
10812464	Sa1158c	ST8	aac3, aac(6'), arlR, arlS, blaI, blaR, blaZ, dhaP, fosB, lmrS, mecA, mepA, mepB, mgrA, norA, norB, rlmH, tet38, tetM, aph(3')	clfA, clfB	fnbA, fnbB	icaA, icaB, icaC, icaD, icaR	spa
11200086	Sa3154	ST351	aac3, aph(3'), arlR, arlS, dhaP, lmrS, mepA, mepB, mepR, mgrA, norA, norB, rlmH, tet38	clfA, clfB	xx	icaA, icaB, icaC, icaD, icaR	xx
40915913	Sa3603	ST352	aac3, aph(3'), arlR, arlS, dhaP, lmrS, lnuA, mepA, mepB, mepR, mgrA, norA, norB, rlmH, tet38	clfA, clfB	fnbA, fnbB	icaA, icaB, icaC, icaD, icaR	spa
40913704	Sa3489	ST352	aac3, aph(3'), arlR, arlS, dhaP, lmrS, lnuA, mepA, mepB, mepR, mgrA, norA, norB, rlmH, tet38	clfB	fnbA, fnbB	icaA, icaB, icaC, icaD, icaR	spa
40913568	Sa3493	ST352	aac3, aph(3'), arlR, arlS, dhaP, lmrS, lnuA, mepA, mepB, mepR, mgrA, norA, norB, rlmH, tet38	clfA, clfB	fnbA, fnbB	icaA, icaB, icaC, icaD, icaR	spa
31312165	Sa3014	ST352	aac3, aph(3'), arlR, arlS, dhaP, lmrS, mepA, mepB, mepR, mgrA, norA, norB, rlmH, tet38	clfA, clfB	fnbA, fnbB	icaA, icaB, icaC, icaD, icaR	spa

### Virulence profile of *S. aureus* isolates

The 43 isolates were cultured on blood agar plates and checked for hemolysis. All the isolates either produced alpha-hemolysin (34/43) (encoded by *hla*) or beta-hemolysin (9/43) (encoded by *hlb*) (Fig. 2c,d). Beta-hemolysis was observed only in the members of ST151 and ST8. Crystal violet assay confirmed biofilm-forming ability in all isolates. Specifically, 23.26% of the isolates showed strong biofilm formation, 55.81% of them were moderate biofilm formers, whereas the rest formed weak biofilms (Fig. 2e,f and Table S5). None of the isolates except Sa16, Sa23, and Sa27 from ST352 and ST151 were strong biofilm formers, whereas, isolates from ST8 and ST2270 formed strong biofilms. Common virulence genes included: two-component leukotoxins, including gamma-hemolysin (Hlg, encoded by *hlgA, hlgB,* and *hlgC*), and leukotoxin D (LukD, encoded by *lukD*). None of the isolates had pyrogenic toxin superantigen (PTSAg) genes except for Sa3154 which contained enterotoxin C, enterotoxin L, and toxic shock syndrome toxin-associated *sec, sell,* and *tsst-1* genes, respectively. Adhesins that are involved in biofilm formation were also identified in all isolates. For instance, fibronectin-binding proteins, fnbA, and fnbB were observed in 60.46% of the isolates. Clumping factor A (clfA), a cell-wall anchored protein was identified in 58.13% of the isolates, whereas, clfB, a fibrinogen-binding adhesin was found in 95.56% of the isolates. Accessory gene regulator (agr) and staphylococcal accessory regulator (sarA) system associated with quorum sensing [11] was identified among the isolates as well. Genes associated with intercellular adhesion such as *icaA*, *icaB*, *icaC*, *icaD*, and *icaR* were evident in all 43 isolates. The presence of the staphylococcal protein A (spa) gene, the product of which plays an important role in colonization and immune invasion [12] was found in 17 isolates including 4 ABR isolates. Ssp serine protease (encoded by *sspA*) that contributes to *in vivo* growth and survivability [13] was identified in all the isolates. The second immunoglobulin-binding protein (Sbi) which is a multifunctional immune invasion factor [14] was observed in 34 isolates. Moreover, all the isolates had bovine immune invasion factors such as serotype 8 capsular polysaccharide (Cap), and adenosine synthase A (AdsA). The list of genes associated with adherence, toxin/enzyme production, and immune invasion is provided in Tables 1, 2, and S6.

**Table 2:** List of the genes associated with toxin and enzyme production, and immune invasion

Isolate ID	Isolate notation	Virulence genes											
		**Toxins**							**Enzyme**		**Immune invasion**		
		**Alpha hemolysin**	**Beta hemolysin**	**Gamma hemolysin**	**Delta hemolysin**	**Enterotoxin-like L**	**Leukotoxin D**	**Toxic shock syndrome toxin**	**Cysteine protease**	**Serine protease**	**Capsule**	**AdsA**	**Sbi**
30704176	Sa3	hly/hla	hlb	hlgA, hlgB, hlgC	hld	xx	lukD	xx	sspB, sspC	sspA	cap8A, cap8B, cap8C, cap8D, cap8E, cap8F, cap8G, cap8H, cap8I, cap8J, cap8K, cap8L, cap8M, cap8N, cap8O, cap8P	adsA	sbi
41704653	Sa9	hly/hla	hlb	hlgA, hlgB, hlgC	hld	xx	lukD	xx	sspB, sspC	sspA	cap8A, cap8B, cap8C, cap8D, cap8E, cap8F, cap8G, cap8H, cap8I, cap8J, cap8K, cap8L, cap8M, cap8N, cap8O, cap8P	adsA	xx
32200324	Sa11	hly/hla	hlb	hlgA, hlgB, hlgC	hld	xx	lukD	xx	sspB, sspC	sspA	cap8A, cap8B, cap8C, cap8D, cap8E, cap8F, cap8G, cap8L, cap8M, cap8N, cap8O, cap8P	adsA	sbi
22200587	Sa14	hly/hla	hlb	hlgA, hlgB, hlgC	hld	xx	lukD	xx	sspB, sspC	sspA	cap8A, cap8B, cap8C, cap8D, cap8E, cap8F, cap8G, cap8L, cap8M, cap8N, cap8O, cap8P	adsA	sbi
21000024	Sa30	hly/hla	hlb	hlgA, hlgB, hlgC	hld	xx	lukD	xx	sspB, sspC	sspA	cap8A, cap8B, cap8C, cap8D, cap8E, cap8F, cap8G, cap8L, cap8M, cap8N, cap8O, cap8P	adsA	sbi
10812464	Sa1158c	hly/hla	hlb	hlgA, hlgB, hlgC	hld	xx	lukD	xx	sspB, sspC	sspA	cap8A, cap8B, cap8C, cap8D, cap8E, cap8F, cap8G, cap8L, cap8M, cap8N, cap8O, cap8P	adsA	sbi
11200086	Sa3154	hly/hla	hlb	hlgA, hlgB, hlgC	hld	sell	lukD	tsst-1	sspB, sspC	sspA	cap8A, cap8B, cap8C, cap8D, cap8E, cap8F, cap8G, cap8H, cap8I, cap8J, cap8K, cap8L, cap8M, cap8N, cap8O, cap8P	adsA	sbi
40915913	Sa3603	hly/hla	hlb	hlgA, hlgB, hlgC	hld	xx	lukD	xx	sspB, sspC	sspA	cap8A, cap8B, cap8C, cap8D, cap8E, cap8F, cap8G, cap8L, cap8M, cap8N, cap8O, cap8P	adsA	sbi
40913704	Sa3489	hly/hla	hlb	hlgA, hlgB, hlgC	hld	xx	lukD	xx	sspB, sspC	sspA	cap8A, cap8B, cap8C, cap8D, cap8E, cap8F, cap8G, cap8L, cap8M, cap8N, cap8O, cap8P	adsA	sbi
40913568	Sa3493	hly/hla	hlb	hlgA, hlgB, hlgC	hld	xx	lukD	xx	sspB, sspC	sspA	cap8A, cap8B, cap8C, cap8D, cap8E, cap8F, cap8G, cap8L, cap8M, cap8N, cap8O, cap8P	adsA	sbi
31312165	Sa3014	hly/hla	hlb	hlgA, hlgB, hlgC	hld	xx	lukD	xx	sspB, sspC	sspA	cap8A, cap8B, cap8C, cap8D, cap8E, cap8F, cap8G, cap8L, cap8M, cap8N, cap8O, cap8P	adsA	sbi

### Internalization of the *S. aureus* isolates in human intestinal epithelial cells

**Fig. 3:**
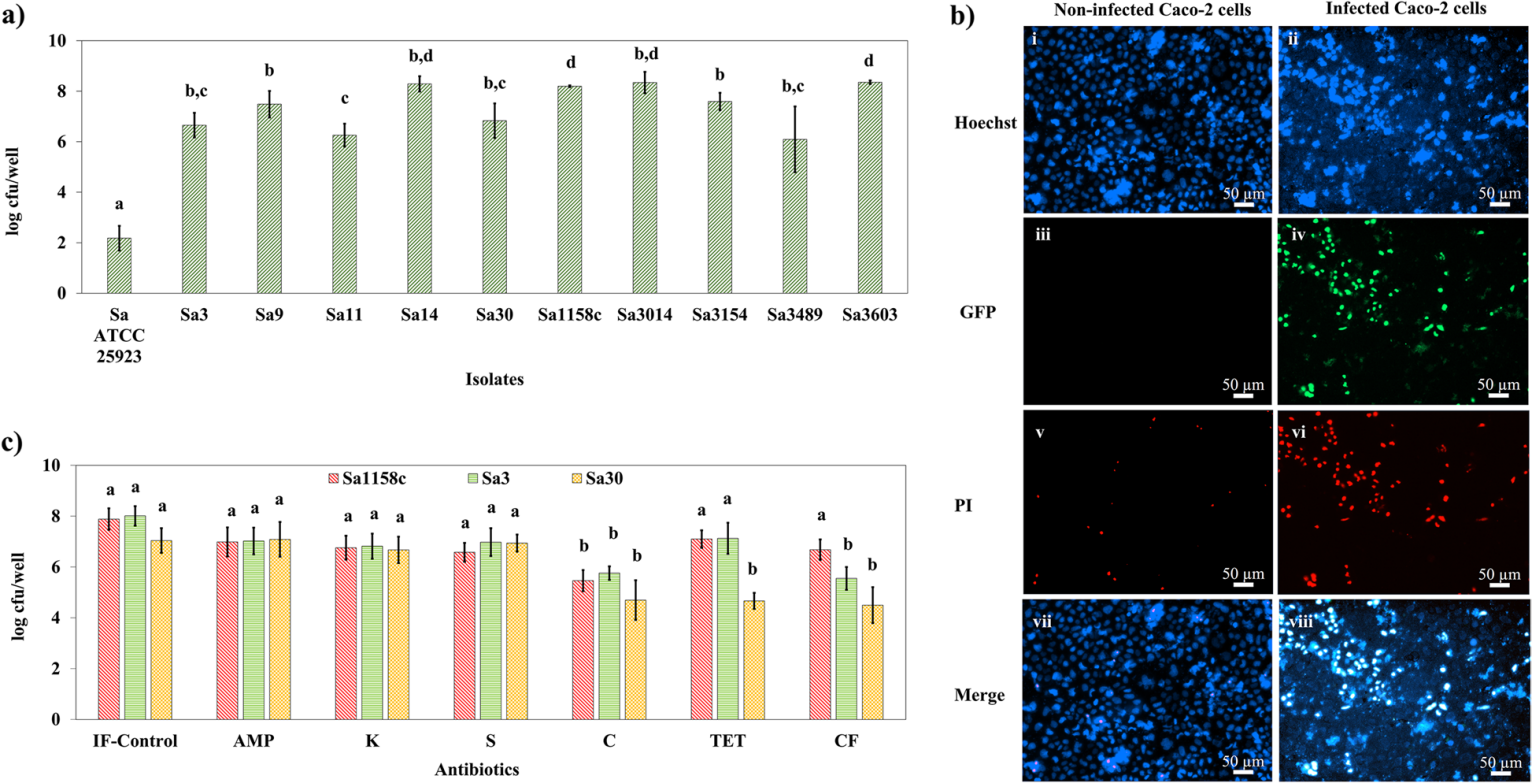
Internalization of *S. aureus* isolates and response to antibiotic treatment in Caco-2 cells. **a** Intracellular survivability of *S. aureus* isolates in Caco-2 cells. Approximately 2 × 10^4^ Caco-2 cells/well of a 96-well plate were exposed to the isolates maintained at 0.5 Macfarland standard (1.5 X 10^8^ cells/ mL). The cells were washed with PBS and subjected to gentamicin (10 µg/mL) to remove extracellular bacteria. The cells were washed further after 4 h of incubation and lysed using 0.5% (v/v) of Triton-X. Colony-forming units were determined using the drop culture method. **b** Epifluorescence microscopic images of non-infected control and Sa30-infected Caco-2 cells. After 4 h incubation of the Caco-2 cells exposed to bacteria, 30 µL of Hoechst-PI cocktail was added and incubated for 30 min in dark. These wells were imaged using an epifluorescence microscope with blue, green, and red filters. i-ii) Hoechst 33342 staining of non-infected and Sa30-infected Caco-2 cells. iii-iv) GFP-labeled Sa30 internalization in Caco-2 cells. Non-infected cells were considered as a control. v-vi) PI staining of infected and non-infected cells. vii-viii) Overlap of Hoechst and PI stained Sa30-infected and non-infected Caco-2 cells. The images confirmed Sa30 internalization and infection of the Caco-2 cells leading to cell death. **c** Antibiotic efficiency against intracellular Sa1158c, Sa30, and Sa3. The infected Caco-2 cells were exposed to ampicillin (AMP) (10 µg/mL), kanamycin (K) (30 µg/mL), streptomycin (S) (10 µg/mL), chloramphenicol (C) (30 µg/mL), tetracycline (TET) (30 µg/mL), and ceftiofur (CF) (30 µg/mL). The plates were incubated for 24 h, followed by washing, lysing of the cells, and drop-culture method to enumerate the cfu/well. Average values plotted in the graph with different alphabets indicate a significant difference (*p* < 0.05). ‘IF-control’ stands for infected Caco-2 cells without antibiotic treatment. The experiment was performed in quadruplicates and repeated thrice to ensure reproducibility

Five antibiotic-resistant isolates (Sa3, Sa9, Sa1158c, Sa3489, and Sa3603), and five antibiotic-susceptible isolates (Sa11, Sa14, Sa30, Sa3014, and Sa3154) were tested for internalization in Caco-2 cells. All the isolates showed significantly (*p* < 0.05) higher internalization in Caco-2 cells in comparison to the reference strain, Sa ATCC 25923 (Fig. 3a). For instance, >8 log_10_ cfu/well of Sa14, Sa1158c, Sa3014, and Sa3603, and >6 log_10_ cfu/well of Sa3, Sa9, Sa11, Sa30, Sa3154, and Sa3489 were recovered from the Caco-2 cells, which was ~5.22 log_10_ higher (*p* < 0.05) than the reference strain. High-content microscopy confirmed the internalization of Sa30 and the death of Caco-2 cells (Fig. 3b(i-viii)). Cysteine proteases, staphopain B (SspB), and staphopain C (SspC) which are often associated with biofilm formation and intracellular colonization of *S. aureus* [15, 16] were identified in all isolates (Tables 1 and S6).

The Caco-2 cells with internalized *S. aureus* were exposed to antibiotics to check for their colonization-remediation efficiency. The aminoglycosides and ß-lactam antibiotics failed to show effectiveness against any of the intracellular *S. aureus* whereas, chloramphenicol, tetracycline, and ceftiofur were comparatively more effective (*p* < 0.05) against all the antibiotic-susceptible and lincosamide resistant isolates. However, none of these antibiotics could reduce the intracellular bacterial load by more than 2.5 log_10_. For instance, chloramphenicol, tetracycline, and ceftiofur showed a 2.3-2.5 log_10_ reduction (*p* < 0.05) of Sa30 colonization (Fig. 3c). Moreover, no antibiotic demonstrated a significant reduction in the aminoglycoside/ß-lactam/tetracycline/cephalosporin-resistant Sa1158c colonization except chloramphenicol which showed a 2.42 log_10_ reduction (*p* < 0.05) (Fig. 3c). Similarly, tetracycline failed to show any efficiency against the tetracycline-resistant Sa3 and Sa9, while chloramphenicol and ceftiofur significantly reduced their colonization by ~2.35 log_10_ (Figs. 3c and S1a).

### Pathogenicity of the *S. aureus *isolates in *Caenorhabditis elegans* model of intestinal infection

**Fig. 4:**
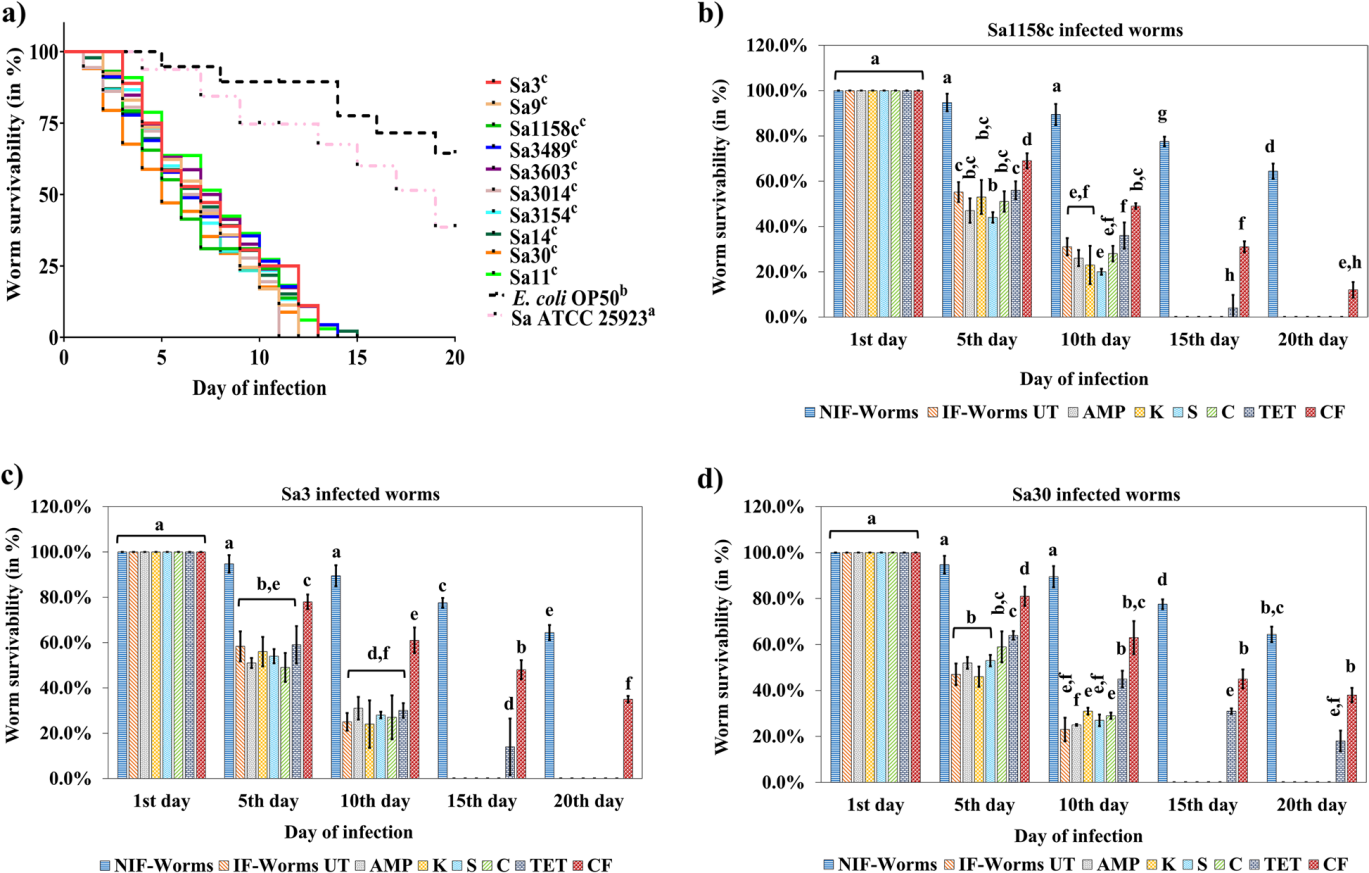
Life-span of *S. aureus* infected *C. elegans* and assessment of antibiotic efficiency. **a** Life-span assay of *S. aureus* infected and non-infected *C. elegans*. The worms were grown to the L4 stage and exposed to 1.5 × 10^8^ cells of *S. aureus* isolates for 48 h. The worms were washed, transferred to NGM plates with *E. coli* OP50 lawns, and monitored for survivability for 20 days. The mantel-Cox test was used to find the statistical significance (p < 0.05). **b**-**d** Antibiotic efficiency against **b** Sa1158c, **c** Sa3, and **d** Sa30 infection in *C. elegans*. The infected worms were subjected to ampicillin (AMP) (10 µg/mL), kanamycin (K) (30 µg/mL), streptomycin (S) (10 µg/mL), chloramphenicol (C) (30 µg/mL), tetracycline (TET) (30 µg/mL), and ceftiofur (CF) (30 µg/mL) for 24 h. The worms were washed and transferred to NGM plates with *E. coli* OP50 lawns. The survivability of the worms was monitored for 20 days. Average values plotted in the graph with different alphabets indicate a significant difference (*p* < 0.05). ‘NIF-Worms’ and ‘IF-Worm UT’ stands for non-infected worms and infected untreated worms, respectively. The experiment was performed in quadruplicates and repeated thrice to ensure reproducibility

**Fig. 5:**
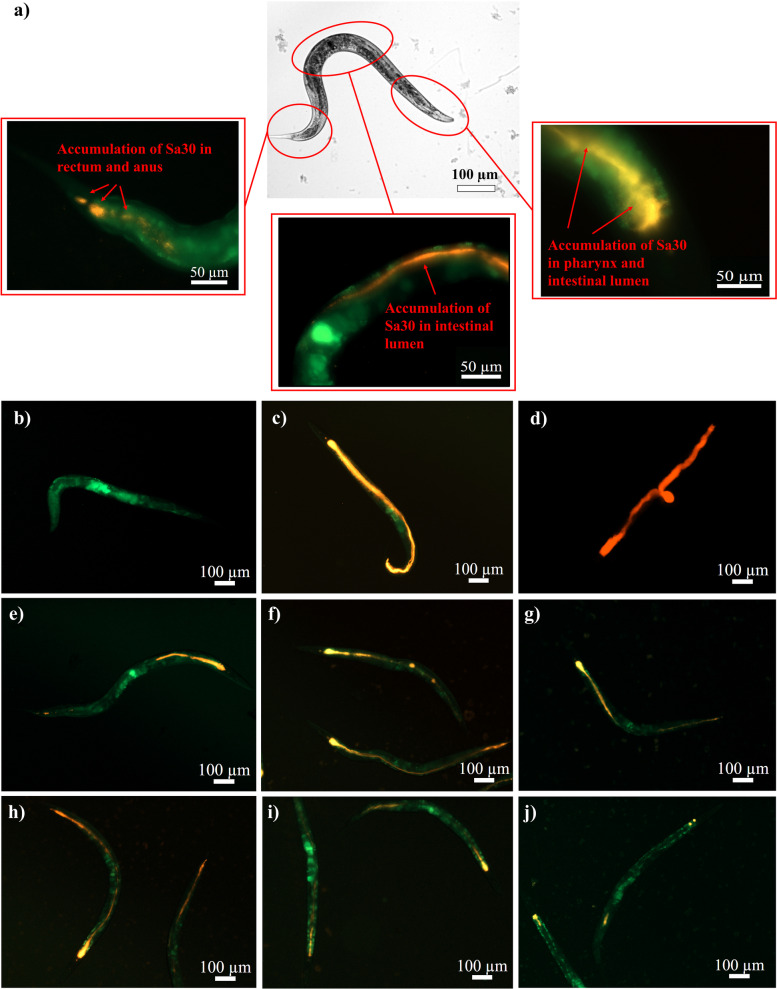
Microscopic images of non-infected, and antibiotic-treated/untreated Sa30-infected *C. elegans*. Epifluorescence images of **a** Sa30 infected (24 h post-infection), **b** non-infected, **c** Sa30 infected (48 h post-infection), and **d** dead infected worms. Sa30 accumulation was observed in the pharynx, intestinal lumen, rectum, and anus of the worms 24 h post-infection. The increased fluorescence 48 h post-infection throughout the digestive tract of the worms suggested Sa30 multiplication leading to intestinal epithelium destruction. The antibiotics **e** ampicillin, **f** kanamycin, **g** streptomycin, **h** chloramphenicol, **i** tetracycline, and **j** ceftiofur were exposed to Sa30 infected worms for 24 h. The worms were washed and resuspended in S-basal media in a 96-well plate. Green (505 nm) and red (583 nm) filter combinations were used to acquire the green autofluorescence of *C. elegans* and RFP-labeled Sa30. The epifluorescence images were captured using the Cell Discoverer 7

Infection of *C. elegans* with the selected isolates had a significant effect on the lifespan of worms when compared with non-infected or infected with reference strain (Sa ATCC 25923). For instance, 100% death in the infected worms was observed by the 15^th^ day post-infection, whereas ~77.5%, and ~60% of the non-infected and Sa ATCC 25923 infected worms respectively, were alive on the 15^th^ day (Fig. 4a). High-content microscopy confirmed Sa30 accumulation in the pharynx, intestinal lumen, rectum, and anus of the worms within 24 h post-infection (Fig. 5a). After 48 h of infection, Sa30 accumulation was observed throughout the digestive tract of *C.* elegans leading to intestinal epithelium destruction, and complete degradation of internal organs (Figs. 5b-d and S2a-i). The multiplication of Sa30 in *C. elegans* was evident with increased fluorescence when the microscopic images of 24 h and 48 h post-infection periods were compared.

The 24 h treatments with ampicillin, streptomycin, kanamycin, and chloramphenicol failed to improve (*p* > 0.05) the lifespan of *C. elegans* infected by any of the 10 selected isolates (Figs. 4b-d and S1b-h). For instance, 20-31% of the worms infected with Sa3 and Sa1158c were alive (*p* > 0.05) irrespective of treatment by these antibiotics on the 10^th^ day (Fig. 4b,c). Tetracycline failed to improve (*p* > 0.05) the survivability of Sa3 and Sa1158c infected worms on the 10^th^ day either, while ceftiofur treatment was comparatively effective (*p* < 0.05) against both these isolates. Only 25-29% of the Sa30 infected worms were alive after ampicillin, streptomycin, kanamycin, and chloramphenicol treatment on the 10^th^ day which was insignificant (*p* > 0.05) when compared to untreated control (Fig. 4d). The low antibiotic efficiency was verified through higher fluorescence showing Sa30 accumulation in the pharynx, intestinal lumen, and anus of the worms (Fig. 5e-h). In comparison, 45% of the Sa30 infected worms were alive after tetracycline treatment, which was supported by low fluorescence in the pharynx, rectum, and anus regions suggesting reduced Sa30 accumulation (Fig. 5i). A significant (*p* < 0.05) improvement in the survivability of Ceftiofur-treated Sa30 (63% until the 10^th^ day) infected worms was observed which was confirmed by a negligible accumulation of the isolate in the pharynx, and rectum regions (Fig. 5j).

## Discussion

A subpopulation of *S. aureus* isolates (n=43) from MPCC was investigated for ABR and virulence characteristics favoring intracellular survival and evasion of antibiotics. All isolates possessed critical virulence characteristics such as biofilm formation, and hemolysis manifestation while ABR phenotype was evident only among six isolates. Genes associated with ABR, toxin production, adherence, and host immune invasion were identified by analyzing the whole-genome sequences. Although none of the isolates carried human adaptation genes, they demonstrated intracellular invasion, infection, and death of human epithelial Caco-2 cells, and intestinal infection model, *Caenorhabditis elegans.* Our studies also showed the inefficiency of widely used antibiotics relevant to veterinary and public health to remediate intracellular and intestinal *S. aureus* infection.

Out of the 43 isolates, 5 isolates (Sa3, Sa9, Sa3489, Sa3493, and Sa3603) showed resistance to either tetracycline or lincomycin, whereas one isolate (Sa1158c) was resistant to multiple antibiotics. Approximately 39.5% and 47.5% of the isolates showed intermediate responses toward lincomycin and spectinomycin, respectively. These two antibiotics have been introduced in Canada to swine, poultry, and dairy cattle individually or in combinations to a large extent [17]. Bennett *et al.* have reported *S. aureus* showing resistance to lincosamides and aminoglycosides frequently and rapidly due to their similar mechanism of action [18]. Sa3 and Sa1158c carried *tetK* and *tetM* genes, respectively while all 43 isolates were identified with the major facilitator superfamily (MFS) of transporters such as *tet(38)*, *NorA*, and *NorB* efflux genes. These chromosomally encoded MFS efflux genes are associated with EtBr extrusion and their expressions are controlled in part by the transcriptional regulators *mgrA* and the *arlRS* [19–22] which were also identified in all isolates. Interestingly, tetracycline resistance (from disc diffusion assay) and EtBr efflux activity were evident only in Sa3, Sa9, and Sa1158c. The role of functional efflux pumps regulated by the MFS transporters in conferring tetracycline resistance in the three isolates (Sa3, Sa9, and Sa1158c) is evidenced by the congruence of the EtBr assay result and the antibiotic disc diffusion tests [23]. While the exact reason for the failure of ABR genes to translate to functional proteins and phenotype was beyond the scope of this investigation, we speculate possibilities of gene mutations, failure in sensing antibiotic stress, defective gene products, *etc* as potential causes of the discrepancy between the genetic makeup of the isolates and their phenotypic characteristics [24, 25]. The ß-lactam and cephalosporin resistance in Sa1158c could be associated with expressive ß-lactamase enzyme activity (50.36 U/mL) and the association of *blaI, blaR,* and *blaZ* genes. Moreover, the *mecA* gene identified in Sa1158c encodes the low-affinity penicillin-binding protein (PBP2a) and also plays a significant role in resistance towards ß-lactam antibiotics, especially methicillin, thus is considered to be a standard for MRSA confirmation [12]. The *aac(6')*, *aac(3’)*, and *aph(3')* genes in Sa1158c encode the aminoglycoside-modifying enzymes that mediate gentamycin and kanamycin inactivation, whereas, the prevalence of *lnuA* gene in Sa3489, Sa3493, and Sa3603 encode lincosamide nucleotidyl transferases leading to lincomycin resistance [26, 27].

Every isolate exhibited crucial virulence characteristics essential for an intramammary and intestinal infection. For instance, all the isolates manifested either alpha or beta-hemolysin and formed biofilms. Hemolysins encoded by *hly/hla*, and *hlb* genes identified in the isolates often contribute to cell signaling pathways that mediate proliferation, cytokine secretion, inflammatory responses, and cell-cell interactions [28]. The role of the Clf-Sdr family consisting of fibrinogen-binding proteins, clfA, and clfB, and fibronectin-binding proteins (FnBPs), fnbA*,* and fnbB in biofilm formation and their contribution to *S. aureus* infection has been extensively discussed [29]. Moreover, the *ica*ADBC locus identified in all the isolates mediates the synthesis of polysaccharide intercellular adhesin (PIA) which is poly *N*-acetyl glucosamine (PNAG), one of the extracellular polymeric substances of staphylococcal biofilms [30]. Here, all the members of ST151 manifested beta-hemolysis, and over 76% of the isolates formed strong or moderate biofilms. ST151 subclone of the bovine ET3 clone has also been reported to display greater virulence in mouse models of mastitis by Guinane *et al.* [31]. ST8 has been reported to be a human clone that has transferred to dairy cows causing mastitis and has been speculated for possible spillover and transmission under suitable selection pressure [1].

All the tested isolates showed intracellular colonization (of >6 log_10_ cfu/well), and infection of the Caco-2 cells causing cell death. The intracellular invasion and infection were possibly mediated by the cysteine proteases SspB, and SspC which were evident in all the isolates [16]. The fibronectin bridging between *S. aureus* FnBPs and the Caco-2 fibronectin receptor integrin α_5_β_1_ is also known to contribute to epithelial cell invasion [32]. Moreover, Kwak *et al*. have reported the contribution of staphylococcal hemolysin in the disruption of the barrier integrity of Caco-2 cells [9]. As reported in this study, the internalized bacteria were able to cause cell death. Accordingly, cytotoxic *S. aureus* strains upon internalization by epithelial cells have the ability to escape from the phagosome, multiply in the cytosol, employ staphylococcal cysteine proteases and induce host cell death [16].

The microscopic images showed Sa30 accumulation in the pharynx, intestine, rectum, and anus of *C. elegans*. The consequence of the infection was evident as a significant reduction in worm survivability. Our observations are consistent with those made by Irazoqui *et al.* where *S. aureus* was reported to cause enterocyte effacement, lysis of the intestinal epithelial cells, followed by invasion in the rest of the body, and complete degradation of the internal tissues of *C. elegans* [33]. The pathogenicity of the isolates in *C. elegans* was probably executed by the production of alpha-hemolysin (encoded by *hla*), and V8 protease (encoded by *sspA*) [34]*.* Moreover, overexpression of the *icaADBC* locus synthesizing PIA is also reported to be one of the contributing virulence mechanisms employed by *Staphylococci* during intestinal infection of nematodes [35]. Sa ATCC 25923 infected worms showed better survivability in comparison to the worms infected by the bovine isolates. This is probably because the reference strain lacks expressive virulent genes and a strong intracellular internalization property which are reported to be essential for *C. elegans* infection.

Different classes of antibiotics including aminoglycosides, cephalosporin, ß-lactam, tetracycline, and chloramphenicol were tested for intracellular and intestinal infection remediation in Caco-2 cells, and *C. elegans*, respectively. The antibiotics were selected based on their frequent use against *S. aureus-*mediated mastitis and human infections. Kanamycin, streptomycin, and ampicillin failed to reduce bacterial colonization in the Caco-2 cells. The result was expected as the conventional antibiotics especially ß-lactams, aminoglycosides, and cephalosporin (cellular/extracellular (C/E) ratio: <1) are known to have poor penetration, slow accumulation, or inferior retention in mammalian cells due to their high polarity and hydrophilic characteristics [36]. Chloramphenicol showed superior efficiency which is probably because of higher accumulation (C/E ratio: 2-4) due to its hydrophobic nature [37]. Tetracycline (C/E ratio: 1.8-7.1) is reported to moderately penetrate and accumulate inside neutrophils through active organic cation transport with a relatively low affinity [38]. Despite being a cephalosporin, ceftiofur showed better efficiency against *S. aureus* infection in Caco-2 cells in comparison to other antibiotics. This could probably be due to a direct antibacterial effect of ceftiofur owing to a higher intracellular concentration above the minimum bactericidal concentration [36]. Although few antibiotics were comparatively effective, none of them reduced bacterial colonization by more than 2.5 log_10_. Our observations of lower antibiotic efficiency against intracellular *S. aureus* corroborate with several existing literature [36, 39, 40]. The other classes of antibiotics that are known for higher C/E ratios such as quinolones, carbapenems, *etc*. are mostly restricted for veterinary use as they are considered to be the last-resort drugs for human applications [41].

Microscopic images indicated a reduction in Sa30 accumulation in *C. elegans* after aminoglycoside, ampicillin, and chloramphenicol treatment, which however was not enough to inhibit Sa30 multiplication leading to infection and cell death as suggested by the life-span assay. Tetracycline and ceftiofur were comparatively effective in remediating *S. aureus-*mediated intestinal infection in the nematodes. This is probably because, in addition to the antimicrobial activity, tetracycline has been reported to up-regulate *C. elegans* genes that function in xenobiotic detoxification, redox regulation, and cytoprotection [42]. Cephalosporins on the other hand have been reported to employ anti-quorum sensing activity, reduce bacterial motility, and biofilm formation [43]. Despite its better performance in Caco-2 cells, chloramphenicol wasn’t effective in infection remediation in worms which is probably because of the impermeability of the worm cuticle to hydrophobic compounds thus leading to inefficient drug uptake [44]. The aminoglycosides and ß-lactam antibiotics failed as well possibly because of their poor penetration against the *S. aureus* internalized in the intestinal epithelial cells of *C. elegans*. Generally, antibiotics that were found effective against bacteria from the Kirby Bauer disc diffusion assay failed to show convincing efficiency in both the infection models. This discrepancy in the effectiveness of antibiotics when directly exposed to bacterial cells and those present as intracellular pathogens originating from differential pharmacokinetics of antibiotics highlight the importance of testing antibiotics in organism models.

Proteins associated with bovine immune invasions such as Sbi, Cap, and AdsA were identified in the isolates. Previous studies have reported Sbi to participate in the inflammatory response induced during staphylococcus infections whereas, Cap and AdsA inhibit neutrophil activity against *S. aureus* thus preventing chemotaxis and phagocytosis and promoting cell adhesion, during pathogen spillover to humans [45–47]. Sa3154 from ST351 was the only isolate to carry PTSAgs which are known to regulate immune responses by abnormal activation of immune cells or exhibit destruction of the host cell membranes [48]. The prevalence of PTSAg genes varies considerably between studies probably due to variations in the herd selection criteria or geographical locations [48]. In this study, the low prevalence of PTSAg gene-positive *S. aureus*, the lack of virulence markers (*lukE*, *lukM*), and human immune evasion cluster-associated genes (*lytN, fmhC, dprA, chp, sak,* and *scn*) suggest a minimal risk of zoonotic infection. However, *S. aureus* is an opportunistic pathogen and thus, under certain environmental pressure, mobile genetic elements may disseminate within or across different lineages [1]. For instance, antibiotic pressure-inducing SOS response has been reported to promote horizontal gene transfer of pathogenicity islands in *Staphylococci* [49, 50].

## Conclusion

*S. aureus* is highly prevalent in bovine mastitis cases worldwide, and thus investigations associated with ABR, ABR mechanisms, and virulence characteristics are crucial. In this study, we used phenotypic and genotypic profiling to inspect 43 *S. aureus* isolates associated with bovine mastitis. Antibiotic resistance was identified among six isolates based on a disc diffusion assay and all the isolates demonstrated crucial virulence characteristics including hemolysis induction, biofilm formation, intracellular infection in Caco-2 cells, and intestinal infection in *C. elegans*. Although these isolates lacked human immune invasion genes, their ability to invade human cells and cause intestinal infection in model organisms highlights the need for continuous monitoring for zoonosis. Notably, the antibiotics that showed efficiency against the isolates in the disc diffusion assay failed to a large extent in remediating their intracellular and intestinal infection. This observation suggested the need to include models capable of testing the effectiveness of antibiotics against internalized bacteria as well as the urgent need to develop therapeutics that can combat ABR and intracellular pathogens. Nano-enabled antibacterial combination therapies designed to deliver multiple drugs hold promise in countering such recalcitrant infections caused by ABR bacteria [51, 52].

## Methods

### Isolation of the *S. aureus* from cases of clinical mastitis

A library of 43 *S. aureus* isolates analyzed in this study are a part of the mastitis pathogen culture collection (MPCC) and collected from different Canadian provinces (Alberta, Ontario, Quebec, and Atlantic provinces) [53, 54]. The metadata including the numbers and locations of the herd, sampling dates, mastitis severity score, isolate IDs, and notations are summarized in Table S1. The isolates were grown in Tryptic Soy Agar (TSA) plates with 5% sheep blood (Hardy Diagnostics, Canada), but were cultured in Mueller-Hinton broth (MHB) (Millipore Sigma, Canada) for conducting assays.

### Susceptibility testing of the isolates against antibiotics

The *S. aureus* isolates were subjected to the Kirby-Bauer disc diffusion susceptibility tests following the clinical and laboratory standard institute (CLSI) guidelines [55]. Briefly, 24 antibiotics (Oxoid, Thermo Fischer Scientific, Canada) relevant to human and veterinary health from the classes of ß-lactams, aminoglycosides, cephalosporins, quinolones, macrolides, lincosamide, tetracycline, chloramphenicol, and sulphonamide were included in the study. The list of antibiotics and their corresponding antibiotic concentration breakpoints suggested by CLSI are provided in Table S2 [55]. *Escherichia coli* ATCC 25922, *Staphylococcus aureus* ATCC 25923, and *Pseudomonas aeruginosa* ATCC 27853 (Oxoid company, Canada) were used as quality control (QC) strains.

### Detection of antibiotic resistance mechanisms in the isolates

Activities of the efflux pump and ß-lactamase enzyme in the isolates were determined as phenotypic characteristics of ABR. The efflux pump activities in the isolates were assessed following a pre-established protocol [56]. Briefly, bacterial cells cultured overnight in MHB were washed twice in phosphate-buffered saline (PBS) (1X) and adjusted to 1.0 McFarland standard (approximately 3 × 10^8^ cfu/mL) using DensiCHEK plus (BioMerieux, USA)). Ethidium bromide (EtBr) (3 µg/mL) was added to the bacterial suspensions followed by 30 µg/mL of an efflux pump inhibitor, chlorpromazine (CPZ). The bacterial suspensions were incubated under shaking at 25 ˚C for an hour to allow maximum intracellular accumulation of EtBr. The suspensions were washed, resuspended in PBS, and transferred (140 µL) to 96-well plates. The cells were reenergized to trigger the efflux of EtBr by adding 10 µL of glucose (0.4% v/v), and the fluorescence was monitored for 60 min at 37 ˚C using a plate reader (SpectraMax-i3X, Molecular Devices, USA) at 530/590 nm (excitation/emission). *S. aureus* ATCC 25923 with no efflux pump activity was used as a reference strain. GraphPad Prism 7 software was used to determine the time-dependent efflux of EtBr using a single exponential decay equation as detailed previously [7]. The time taken for the bacterial cells to extrude 50% of EtBr was denoted as t_efflux50 %_.

The Nitrocefin assay was performed to determine the ß-lactamase enzyme activity [56]. For this, isolates grown overnight in MHB were adjusted to McFarland 1.0. Ampicillin (5 µg/mL and 25 µg/mL for ampicillin susceptible and resistant isolates, respectively) was added to the cell suspensions and incubated for 3 h at 37°C. Subsequently, suspensions were centrifuged at 8,900 × g for 10 min and the cells were washed in sodium phosphate buffer (pH – 7.0). Cells resuspended in buffer were sonicated on ice for 3 mins and the cell-free extract was collected by centrifugation (17,500 × g for 25 min). Ten microlitres of Nitrocefin (abcam, Canada) (stock concentration: 0.5 mg/mL) were added to 10 µL of the cell-free extract in a 96-well plate. The final volume was adjusted to 100 µL using the buffer. The absorbance was recorded in kinetic mode for 15 min at 490 nm using a plate reader. *S. aureus* ATCC 25923 with no ß-lactamase enzyme activity was used as a reference strain. The β-lactamase enzyme activity was calculated using the formula:

$$\beta\text{- Lactamase enzyme activity}=\frac{S_a}{\text{Reaction time X }{\text{S}}_\text{v}}$$

where S_a_ is the amount of nitrocefin (in μM) hydrolyzed between t_1_ and t_2_ of the standard curve; reaction time is the time difference between t_1_ and t_2_, and S_v_ is the sample volume (in mL) added to the well. The β-lactamase activity was reported as U/mL.

### Determination of virulence characteristics in the isolates

The isolates were tested for hemolysin activity by growing colonies on Tryptic Soy Agar (TSA) plates containing 5% sheep blood. These plates were incubated for 24 h at 37 ˚C. The pattern of hemolysis was detected by visual inspection for the translucency around the bacterial colony as detailed previously [57].

Biofilm-forming abilities of isolates were assessed by the crystal violet assay [58]. Briefly, 10 µL of bacterial culture maintained at 0.5 McFarland standard (approximately 1.5 × 10^8^ cfu/mL) was added to 100 µL of MHB media in a 96-well plate. The plates were incubated for 24 h at 37 °C without shaking. After incubation, the media was discarded, and the wells were washed with saline to remove non-adherent cells. These plates were kept undisturbed to fix the biofilms at room temperature for 15 min after adding 100 µL of methanol (99% v/v) to each well. The wells were air-dried, followed by the addition of 200 µL of crystal violet (0.4% v/v) and incubation for 2 h. Wells in these plates were washed with saline and 100 µL of acetic acid (30% v/v) was added. The biofilm biomass was quantified by detecting absorbance at 570 nm. The isolates were classified into non-biofilm-formers, weak biofilm-formers, moderate biofilm-formers, and strong biofilm-formers by using the following formulae: OD_cut−off_ = OD_avg_ of control + 3 × standard deviation (SD) of ODs of control; OD ≤ OD_cut−off_ = Non-biofilm-former (NBF); OD_cut−off_ < OD ≤ 2 × OD_cut−off_ = Weak biofilm-former (WBF); 2 × OD_cut−off_ < OD ≤ 4 × OD_cut−off_ = Moderate biofilm-former (MBF); OD > 4 × OD_cut−off_ = Strong biofilm-former (SBF) [7]. *S. aureus* ATCC 25923 was used as a reference strain.

### Evaluation of intracellular survival of *S. aureus* isolates in human intestinal epithelial cells

Cellular internalization of the isolates and the response of internalized bacteria to antibiotics were determined as previously described [56]. Caco-2 cells (ATCC, Virginia, USA) was used as the *in vitro* model of the human intestinal epithelium. The cells were cultured in a 96-well plate (2 × 10^4^ cells/well) until confluent. Five antibiotic-susceptible and five resistant isolates were randomly selected, and cultured overnight. The bacterial isolates (1.5 X 10^8^ cells/mL) were added to each well to infect the Caco-2 cells and the plate was incubated for an hour. The cells were washed using PBS (4 ˚C) and subjected to gentamicin (10 µg/mL) for 30 min. The extracellular gentamicin was removed by washing the cells with PBS followed by incubation with Gibco Dulbecco's Modified Eagle Medium (DMEM) (Thermofisher, Canada) for 4 h to establish the intracellular infection model. Six antibiotics relevant to animal and human infection including ampicillin (10 µg/mL), kanamycin (30 µg/mL), streptomycin (10 µg/mL), chloramphenicol (30 µg/mL), tetracycline (30 µg/mL), and ceftiofur (30 µg/mL) (the standard concentration breakpoints of antibiotics suggested by CLSI) were selected to check their efficiency against intracellular isolates. The plates were incubated for 24 h after the addition of antibiotics in a cell culture incubator at 37 °C, with 5% CO_2_. Subsequently, the cells were washed using PBS and lysed using 0.5% (v/v) of Triton-X. Colony-forming units (cfu) of viable intracellular bacteria were enumerated using the drop culture method as detailed earlier [59]. *S. aureus* ATCC 25923 was used as a reference strain and cells infected with bacteria but without any treatment were considered as the negative control.

### Evaluation of *S. aureus *pathogenicity in *Caenorhabditis elegans* model of intestinal infection

The *C. elegans* CF512 worms were age-synchronized with an alkaline bleach solution to the first larval stage (L1) and further attained the fourth larval (L4) stage with lawns of *E. coli* OP50 on solid nematode growth medium (NGM) for 48 h at 21 °C [60]. These worms were washed with M9 buffer and transferred to NGM plates with lawns of 1.5 × 10^8^ cells of *S. aureus* isolates to establish infection. The plates were incubated for 48 h at 25 ˚C. The infected worms were then washed, resuspended in S-basal media, and exposed to antibiotics in a 96-well plate. After 24 h, the infected worms were washed again and transferred (n=15-30) to fresh solid NGM plates with *E. coli* OP50 lawns. The plates were examined every 24 h for 20 days for worm survivability using a dissection microscope (Wild Heerbrugg, Switzerland). Untreated infected and non-infected worms were considered as controls. Live worms in the plates were scored with a transfer pick.

### Fluorescence microscopic analysis of *S. aureus* in Caco-2 cells and *Caenorhabditis elegans*

Cells of *S. aureus* 30 (Sa30) were transformed with plasmids pSGFPS1 (coding GFP) and pSRFPS1 (coding RFP) to visualize bacterial survival in Caco-2 cells and the *C. elegans* models, respectively. GFP-labeled Sa30 was used to establish infection in Caco-2 cells and avoid interference in the assessment of cell viability, while RFP-labeled Sa30 was used to infect C*. elegans* and avoid interference from green autofluorescent worms. For this, plasmids of pSGFPS1 (BEI resources, NR-51163) and pSRFPS1 (BEI resources, NR-51164) from *S. aureus* RN4220 were purified using the Monarch® Plasmid Miniprep Kit (NEB) after pre-treating with 20 µg of lysostaphin (Sigma-Aldrich) for 30 min at 37 °C [61]. The electrocompetent cells of *S. aureus* 30 (Sa30), originally isolated from mastitic cattle, were prepared as reported earlier [61]. Briefly, 0.1 µg of the purified plasmid DNA and 70 µL of the *S. aureus* competent cells were combined and pulsed at 2.3 kV, 100 Ω, and 25 µF in 0.1 cm cuvette using the Gene Pulser Electroporation System (Bio-Rad Laboratories). The pulsed cells were transferred to 1 mL of brain heart infusion broth and incubated for 1 h at 37 ˚C under constant shaking (5 × g). The cell suspensions were cultured on Luria-Bertani agar (containing 10 µg/mL of trimethoprim) and then incubated overnight at 37 ˚C.

Transformations of bacterial cells were confirmed by acquiring epi-fluorescence images of the bacterial cells using a high-content screening microscope, Cell Discoverer 7 (Carl Zeiss, Germany). The cell death in Caco-2 ensuing bacterial (Sa30) infection was assessed using fluorescence probes. Briefly, the cells were added with 30 µL of fluorescence probes in saline containing Hoechst 33342 (1 μM) (ThermoFisher, Canada) and Propidium iodide (PI) (5 μM) (ThermoFisher, Canada) to image for the number of live and dead cells [59, 62]. These plates were kept at 37 °C for 30 min in the dark and epi-fluorescence images of the Caco-2 cells were captured using channels of blue (Hoechst (461 nm), staining cell nucleus), green (GFP from Sa30), and red (PI (615 nm) staining nucleus of dead Caco-2 cells). Similarly, images of infected and non-infected *C. elegans* were also acquired by Cell Discoverer 7. Green (505 nm), and red (583 nm) filter combinations were used to image green autofluorescence from *C. elegans* and red fluorescence from RFP labeled Sa30.

### Identification of antibiotic resistance, and virulence genes through whole-genome analysis

The extraction and quantification of DNA of each isolate, DNA library preparation, whole-genome sequencing, assembly, and annotation of sequenced reads were conducted as reported previously (Table S1) [1, 54, 63]. Initially, the isolates were identified using matrix-assisted laser desorption ionization-time of flight (MALDI) mass spectrometry. A single well-isolated colony from the TSA plates was cultured overnight at 37°C with agitation. An aliquot of the liquid culture was used for DNA extraction with the DNAzol reagent (Invitrogen) and lysostaphin (Sigma-Aldrich) following the manufacturer’s instructions. Briefly, the Nextera Flex DNA library preparation kit (Illumina, San Diego, CA) and Nextera DNA CD indexes (96 indexes, 96 samples) were used to prepare sequencing libraries as paired-end libraries. The libraries were then sequenced using a MiSeq benchtop sequencer (Illumina) (301 cycles in each direction). The raw DNA sequences of the isolates were assembled using ProkaryoteAssembly version 0.1.6 (https://github.com/bfssi-forest-dussault/ProkaryoteAssembly), and the quality of the genome assemblies was assessed using Qualimap (v. 2.2.2) [7]. Default parameters were applied except for the trimming step for which command trimq=20 was used to trim low-quality sequences (Q score of <20). The sequence types (STs) of each isolate were identified using the tool MLST (v. 2.23.0) (https://github.com/tseemann/mlst) which incorporates data from the PubMLST database. The antibiotic-resistant genes were identified using ABRicate (https://github.com/tseemann/abricate) through the MEGARes database whereas, the genes associated with virulence were assessed using VFanalyzer (http://www.mgc.ac.cn/VFs/main.htm) [1, 57]. Whole genome sequencing data were deposited in BioProject numbers PRJNA609123 (https://www.ncbi.nlm.nih.gov/bioproject/PRJNA609123) and PRJNA622791 (https://www.ncbi.nlm.nih.gov/bioproject/?term=PRJNA622791). The accession numbers for each genome are reported in Table S1.

## Supplementary Information

**Additional file 1: Figure S1.** a) Intracellular responses of Sa9, Sa11, Sa14, Sa3014, Sa3154, Sa3489, and Sa3603 to antibiotic treatment. The aminoglycosides and ampicillin failed to show efficiency against the Caco-2 internalized isolates, whereas chloramphenicol and ceftiofur were comparatively more effective (*p* < 0.05). Tetracycline was more efficient (*p* < 0.05) against all the isolates except the tetracycline-resistant Sa9. b-h) Assessment of antibiotic efficiency against b) Sa9, c) Sa11, d) Sa14, e) Sa3014, f) Sa3154, g) Sa3489, and h) Sa3603 infection in *C. elegans*. The infected worms were exposed to ampicillin (AMP) (10 µg/mL), kanamycin (K) (30 µg/mL), streptomycin (S) (10 µg/mL), chloramphenicol (C) (30 µg/mL), tetracycline (TET) (30 µg/mL), and ceftiofur (CF) (30 µg/mL). The aminoglycosides, ampicillin, and chloramphenicol failed to show infection remediation in the worms, whereas, ceftiofur was comparatively more effective (*p* < 0.05). Tetracycline was effective (*p *< 0.05) as well except against Sa9 infected worms. Average values plotted in the graph with different alphabets indicate a significant difference (*p* < 0.05). ‘IF-control’ stands for infected Caco-2 cells without antibiotic treatment. ‘NIF-Worms’ and ‘IF-Worm UT’ stands for non-infected worms and infected untreated worms, respectively.

**Additional file 2: Figure S2.** Microscopic images of non-infected, and antibiotic-treated/untreated Sa30 infected *C. elegans *under transmission light. Images of a) non-infected, c) Sa30 infected (48 h post-infection), and d) dead infected worms. Destruction of intestinal epithelium and degradation of internal organs was observed due to Sa30 infection. The antibiotics e) ampicillin, f) kanamycin, g) streptomycin, h) chloramphenicol, i) tetracycline, and j) ceftiofur were exposed to Sa30 infected worms for 24 h. The images were acquired using the Cell Discoverer 7.

**Additional file 3: Table S1.** Metadata of the 43 *S. aureus* isolates. **Table S2.** List of antibiotics and antibiotic concentration breakpoints. **Table S3.** List of a) responses of *S. aureus* isolates towards antibiotics, and b) prevalence of antibiotic resistant mechanisms. **Table S4.** List of genes associated to antibiotic resistant mechanisms in *S. aureus* isolates. **Table S5.** List of *S. aureus* isolates with hemolysin production and biofilm formation ability. **Table S6.** List of genes associated to virulence characteristics in the *S. aureus* isolates.

## Data Availability

All supporting datasets have been deposited online. The raw sequence reads have been deposited in the NCBI Sequence Read Archive under BioProject accession numbers PRJNA609123 (https://www.ncbi.nlm.nih.gov/bioproject/PRJNA609123) and PRJNA622791 (https://www.ncbi.nlm.nih.gov/bioproject/?term=PRJNA622791). The accession numbers for each genome are provided in Table S1. The datasets used and analyzed during the current study are available from the corresponding author upon reasonable request.
