# Supplementary figures and images for: Genomic and phenotypic profiling of Staphylococcus aureus isolates from bovine mastitis for antibiotic resistance and intestinal infectivity
Source: BMC Microbiol. 2023 Feb 20;23:43. doi: 10.1186/s12866-023-02785-1 (PMC9940407; doi:10.1186/s12866-023-02785-1)

a)

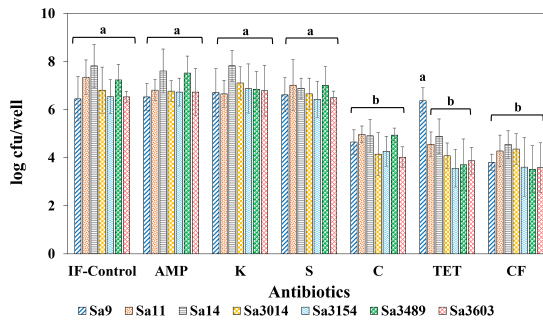

b)

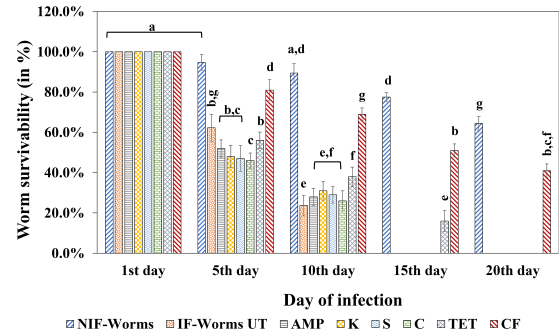

c)

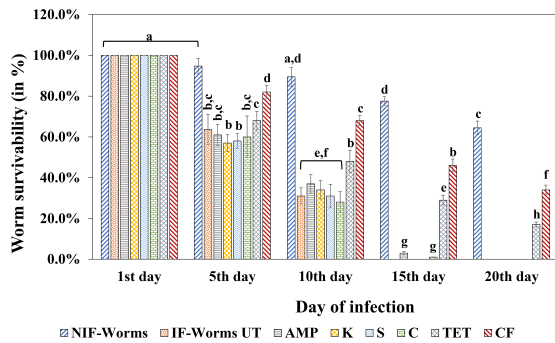

d)

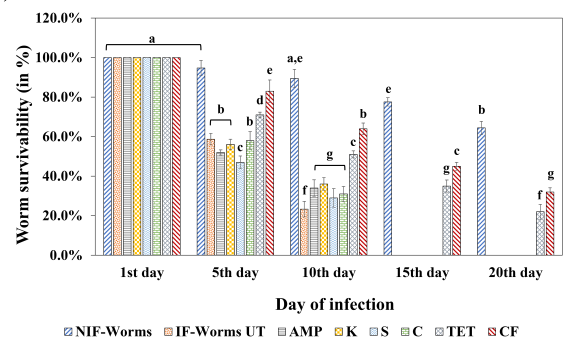

e)

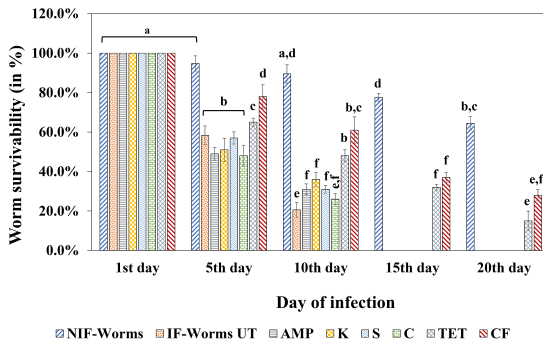

f)

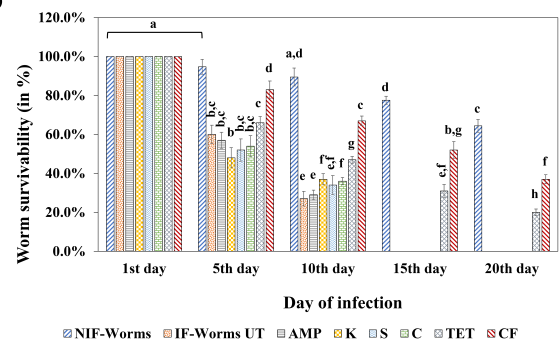

g)

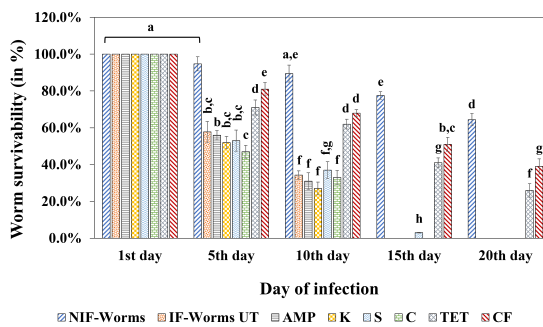

h)

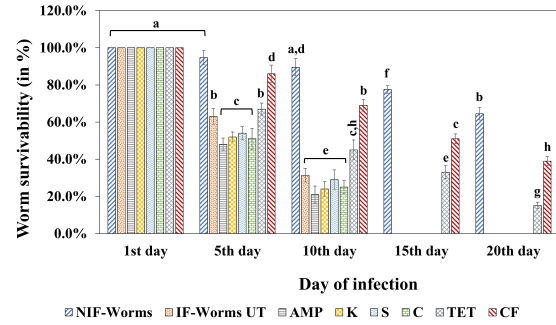

Supplement: Supplementary file 1 — Additional file 1: Figure S1. a) Intracellular responses of Sa9, Sa11, Sa14, Sa3014, Sa3154, Sa3489, and Sa3603 to antibiotic treatment. The aminoglycosides and ampicillin failed to show efficiency against the Caco-2 internalized isolates, whereas chloramphenicol and ceftiofur were comparatively more effective (p < 0.05). Tetracycline was more efficient (p < 0.05) against all the isolates except the tetracycline-resistant Sa9. b-h) Assessment of antibiotic efficiency against b) Sa9, c) Sa11, d) Sa14, e) Sa3014, f) Sa3154, g) Sa3489, and h) Sa3603 infection in C. elegans. The infected worms were exposed to ampicillin (AMP) (10 µg/mL), kanamycin (K) (30 µg/mL), streptomycin (S) (10 µg/mL), chloramphenicol (C) (30 µg/mL), tetracycline (TET) (30 µg/mL), and ceftiofur (CF) (30 µg/mL). The aminoglycosides, ampicillin, and chloramphenicol failed to show infection remediation in the worms, whereas, ceftiofur was comparatively more effective (p < 0.05). Tetracycline was effective (p < 0.05) as well except against Sa9 infected worms. Average values plotted in the graph with different alphabets indicate a significant difference (p < 0.05). ‘IF-control’ stands for infected Caco-2 cells without antibiotic treatment. ‘NIF-Worms’ and ‘IF-Worm UT’ stands for non-infected worms and infected untreated worms, respectively. [file 12866_2023_2785_MOESM1_ESM.pdf]

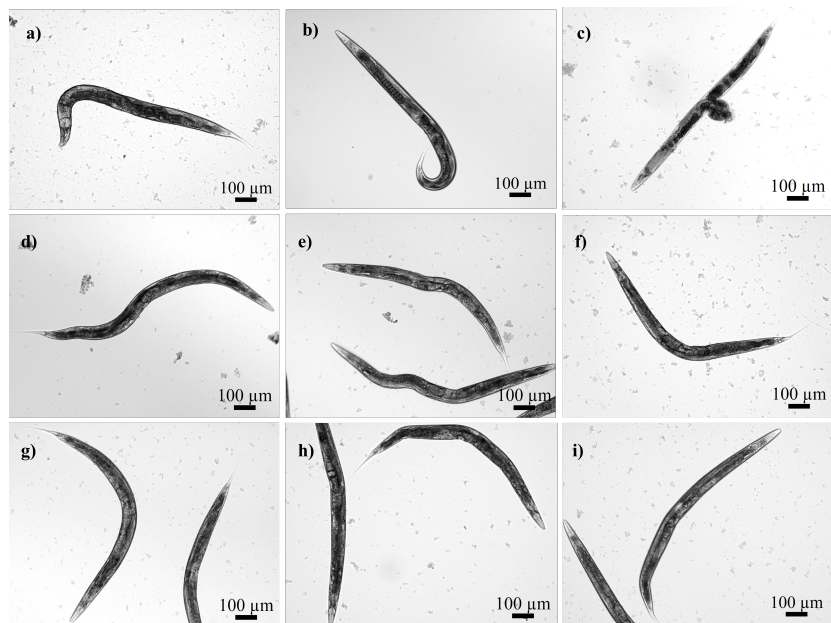

Supplement: Supplementary file 2 — Additional file 2: Figure S2. Microscopic images of non-infected, and antibiotic-treated/untreated Sa30 infected C. elegans under transmission light. Images of a) non-infected, c) Sa30 infected (48 h post-infection), and d) dead infected worms. Destruction of intestinal epithelium and degradation of internal organs was observed due to Sa30 infection. The antibiotics e) ampicillin, f) kanamycin, g) streptomycin, h) chloramphenicol, i) tetracycline, and j) ceftiofur were exposed to Sa30 infected worms for 24 h. The images were acquired using the Cell Discoverer 7. [file 12866_2023_2785_MOESM2_ESM.pdf]
